# *In vitro* and *in vivo* activity of the essential oil and nanoemulsion of *Cymbopogon flexuosus *against *Trichomonas gallinae*

**Published:** 2021

**Authors:** Bruna Baccega, Yan Wahast Islabão, Alexia Brauner de Mello, Filipe Obelar Martins, Carolina Caetano dos Santos, Aline Ferreira Ourique, Samanta da Silva Gündel, Marcia Raquel Pegoraro de Macedo, Élvia Elena Silveira Vianna, Nara Amélia da Rosa Farias, Camila Belmonte Oliveira

**Affiliations:** 1 *Department of Microbiology and Parasitology, Federal University of Pelotas, Pelotas, RS, Brazil*; 2 *Laboratory of Nanotechnology, Franciscan University, Santa Maria, RS, Brazil*

**Keywords:** Phytotherapy, Nanotechnology, Birds, Protozoa, Lemon grass

## Abstract

**Objective:**

This study was done to evaluate the *in vitro* and* in vivo* effects of the essential oil (OE-CL) and nanoemulsion (N-CL) of *Cymbopogon flexuosus* against *Trichomonas gallinae.*

**Materials and Methods:**

*In vitro* assays were done with 10^6^ parasites and OE-CL and N-CL in the concentrations: 110, 220, 330, 440, 550, 660, 770 and 880 µg/ml and four controls: CN (culture medium and trophozoites), MTZ (trophozoites plus 800 µg/ml of metronidazole), TW (trophozoites plus vehicles used for solubilization of derivatives (0.01% Tween) and NB (blank nanoemulsion 880 µg/ml). The *in vivo* assay was done in 35 quails (*Coturnix coturnix*) infected experimentally 4x10^4^ mg/kg, were divided in seven groups (n=5): A (control–healthy), B (control infected), C (control TW 0.01%), D (NB 0.88 mg/kg), E (drug MTZ 25 mg/kg, F (OE-CL at 0.55 mg/kg) and G (N-CL at 0.44 mg/kg), during 7 consecutive days.

**Results:**

The *in vitro* test showed that the OE-CL (550 μg/ml) and N-CL (440 μg/ml) concentrations reduced the trophozoites viability in 100%. In the *in vivo* test, the treatment with OE-CL was efficient on the 4^th^ treatment day and the N-CL after the 3^rd^ day, and the MTZ in the therapeutic concentration was efficient on the 7^th^ day.

**Conclusion:**

It can be observed in this study that the lemon grass has natural potential antitrichomonal activity against *T. gallinae in vitro *and *in vivo*.

## Introduction

*Trichomonas gallinae* is a eukaryotic organism, belonging to the Protist Kingdom (Gaspar da Silva et al., 2007). The infection by the flagellated protozoan, affects primarily the upper digestive and respiratory tract of birds (Columbiformes, Falconiformes, Psittaciformes, Passeriformes and Galliformes) (Forrester and Foster, 2009). Occurring in sylvan birds, as well as in captivity, being which has pigeons (*Columba livia*) as responsible for its distribution, one of its main hosts. (Forrester and Foster, 2009; Bunbury et al., 2007).

 For the treatment of this parasitoses, nitroimidazoles such as metronidazole and tinidazole are used, however, the treatment features therapeutic failures like adapted protocols and resistance of isolates and strains (Dunne et al., 2003; Upcroft et al., 2001; Dingsdag and Hunter, 2018, Zimre Grabensteiner et al., 2011 and Cudmore et al., 2004). Besides, these active principles can present toxicity, and administration of drugs in long-term treatment has carcinogenic potential, possibly due to the relation with DNA, proteins and cell membranes (Hussein et al., 2011; Rein, 1995; Tracy and Webster, 1996).

 New alternatives are necessary to provide a substitute treatment against this protozoan as the use of essential oils (OEs) (Seddiek et al., 2011; Tabari et al., 2017; Youseffi et al., 2017) and the Cymbopogon genus, the *C. flexuosus* botanical species known as lemon grass, is a plant that produces high levels of OE when cultivated in tropical and subtropical regions worldwide and it has been gaining prominence in the use against parasitoses like *Leishmania* (L.) *chagasi*, and *Trypanosoma cruzi* (Ganjewala and Luthra, 2010; Santoro et al., 2007; Oliveira et al., 2012). OEs can suffer environmental variations and the use of nanostructured systems is an efficient strategy to prevent its degradation, improving the beneficial effects and the release of active compounds (Fang et al., 2010; Hosseini et al., 2013). In this context, this study had the objective to investigate the *in vitro* and *in vivo* *Trichomonas *activity in quails (*Coturnix coturnix* japonica) of the essential oil and *Cymbopogon flexuosus* (CF) nanoemulsion.

## Materials and Methods

**Reagents**

CF was commercially acquired from FERQUIMA Indústria e Comércio Ltda. Polysorbate 80 was acquired from Synth (Brazil), Sorbitan monooleate, MTZ (metronidazole) and meio de cultivo utilizado foi trypticase-extrato de levedo-maltose (TYM) was obtained from Sigma-Aldrich^®^, Deisenhofen, Germany and sterile adult bovine serum (SBA), from Bio Nutrients. 

**Development and characterization of nanoemulsion**

The OE-CL and N-CL were supplied by the Nanotechnology Laboratory of the Universidad Fransciscana (UFN). The characterization OE-CL followed the method described by Hussain et al. (2010) with modifications (Gündel et al., 2018), using the Varian Star 3400CX gas chromatograph (CA, USA). For the qualitative analysis of the compounds, a Shimadzu QP2010 Plus gas chromatograph coupled to a mass spectrometer (GC/MS, Shimadzu Corporation, Kyoto, Japan) was used (Gündel et al., 2018).

**Development of nanoemulsion**

The N-CL, oil was developed using the homogenization under high agitation method, following the methodologies described by Gündel et al. (2018). The formulations were composed of an oil phase containing OE-CL (5%) and sorbitan monooleate surfactant (2%), while the aqueous phase was composed of polysorbate 80 (2%) and ultrapure water. Both phases were homogenized separately with the aid of a magnetic stirrer, then the aqueous phase was placed in the Ultra-Turrax® (IKA, Germany) equipment for 10 min at 10,000 rpm. Subsequently, the oil phase was injected into the aqueous phase, and maintained in the Ultra-Turrax® for 30 min at 17,000 rpm, with temperature control. The blank nanoemulsion was developed using a medium chain triglyceride, derived from caprylic and capric acids (Gündel et al., 2018).

**Characterization of nanoemulsion**

The physicochemical characterization of the formulation was done by determining the mean droplet size, polydispersity index, zeta potential and pH. The mean droplet size and polydispersity index were determined by the dynamic light scattering technique (Zetasizer® equipment, nano-ZS model ZEN 3600, Malvern) after sample dilution (500 times) in ultrapure water. The zeta potential was determined using the electrophoretic mobility technique (Zetasizer® equipment, nano-ZS model ZEN 3600, Malvern) after sample dilution (500 times) in aqueous solution of sodium chloride (10 mM). The pH was determined using a potentiometer (DM-22, Digimed®) previously calibrated with standard solution, and the readings were carried out directly in formulations. The formulation of the N-CL remained stable under refrigeration for up to 90 days. The readings were done in triplicate and the results are expressed as mean±standard deviation (Gündel et al., 2018).

***Trichomonas gallinae***

Samples of *T. gallinae *were recovered by the wet mount method of naturally infected pigeons. Twelve native pigeons (*C. livia*) (2 to 8 weeks old) were captured in their nests in the city of Pelotas, Rio Grande do Sul.

Using swabs, samples were taken from the oral cavity, and from membranous lesions of the oropharyngeal region of domestic pigeons (*C. livia*). The culture of the parasite was prepared by immersion of oral swabs in tryptone/ yeast extract/maltose médium (TYM) (Diamond, 1957) supplemented with 10% adult fetal serum, antibiotic (meropenem), antifungal (amphotericin B) (Sigma-Aldrich^®^) and incubated at 37°C (Sansano et al., 2009). The cultures were then examined under an optical microscope at (100 and 400x) for mobile trophozoites observation. 

Cultures were observed over seven consecutive days to verify the growth of trophozoites. At every 48 hr, trophozoites that had more than 95% mobility and normal morphology, were subculture (Seddiek et al., 2014).

**Molecular analyses**

To confirm the *Trichomonas* specie, we did molecular analyses and construct the phylogenetic tree of genera. For the extraction of total genomic DNA, the culture medium sample from the *in vitro* test of *T. gallinae *was used, applying a commercial DNA- GenElute Mammalian Genomic DNA Miniprep (Sigma-Aldrich, St. Louis, MO, EUA) according to the manufacturer's instructions.

To verify the parity of the *T. gallinae* DNA sample, Trichomonad small subunit (SSU) rRNA primers (forward–TACTTGGTTGATCCTGCC and reverse- TCACCTACCGTTACCTTG) and the ITS-1 rDNA sequences were amplified by PCR using the ITS1R-ITS1F and primers were modified according to Cepicka et al (2005). The PCR reaction was run in the final 25 μl volume using Promega PCR Master Mix (Promega Corporation, WI, USA), 20 pM of each primer, and 100-150 ng DNA template. The amplification comprises a denaturation step of 5 min to 95 ^o^C hence of 40 repeats of 60 sec at 95^ o^C, 30 sec at 56^o ^C and 30 sec at 72^o ^C and a final extent at 72^o ^C for 10 min. A positive control (*Caracara plancus*) and a negative control sample were used for each amplification reaction. The amplicons were verified by agarose gel electrophoresis (2%) and observed by staining with Blue Green 1X DNA loading dye (LGC Biotechnology, São Paulo, Brazil). The amplification products were purified using the Illustra GFX (GE Healthcare) PCR and gel DNA purification kit and the DNA strand was sequenced directly (Macrogen; http://www.macrogen.com). The sequence results were matched with the NCBI/Genbank database using the Basic Local Alignment Search Tool (BLASTn). The SSU sequences were used to construct the phylogenetic tree, in software MegaX (Kumar et al, 2018). The phylogenetic trees were inferred using the Neighbor-Joining method and Maximum Likelihood. The robustness of the trees was evaluated using bootstrapping with 1000 replicates. 

***In vitro*** **assay**

To examine the susceptibility of *T. gallinae *to free and nanostructured oils, sterile 96-well plates were used for incubation with different concentrations of OE free and nanostructured. The N-CL was applied to the *in vitro* assay after seven days of the preparation of its formulation.

The parasites were seeded at an initial density of 10^6^ trophozoites/ml of TYM and incubated with the oil and N-CL. Four controls were considered: A (trophozoites only), B (trophozoites plus the vehicle used for solubilization of the derivatives (0.01% Tween (TW), C (trophozoites plus 880 µg/ml metronidazole (MTZ), as positive control) and D (blank nanoemulsion (NB) 880 µg/ml).

Free OE-CL and nanostructured were added to the wells to obtain final concentrations of 110, 220, 330, 440, 550, 660, 770 and 880 μg/ml, respectively. Subsequently, to generate anaerobic conditions, the microculture plates were incubated at 37°C with 5% CO_2 _for 24 h. After this period, a preparation with trophozoites, containing trypan blue (0.4%) (1:1), was evaluated in Neubauer chamber. Cultures with viability equal to or greater than 95% were used for assays, having considered motility, morphology and exclusion by dye.

The IC_50_ (half the maximal inhibitory concentration) was determined for the oil and N-CL. A kinetic growth curve was constructed to obtain the profile of comparable activity of OE and N-CL compounds against *T. gallinae* trophozoites.

Only the concentrations of OE and/or N-CL that showed a reduction in the viability of the trophozoites to 100%, were used to determine the MIC (minimum inhibitory concentration). The trophozoites used to establish MIC and below and above concentrations, as well as inoculated controls in fresh TYM medium at 37°C, were counted in Neubauer chamber with trypan blue every 24 hr for 96 hr to confirm MIC.

The best concentration, presented after MIC free oil analysis and N-CL OEs, was conduted at the following times: 1, 6, 12, 24, 48, 72 and 96 hr by the dye exclusion method. The IC_50_ was determined at varying concentrations, as described in the MIC method. A death time curve was constructed to obtain an activity profile of the efficiency of the free oil and N-CL against *T. gallinae* flagellate trophozoites. All assays were performed independently in nonoplicate (Sena- Lopes et al., 2017).

**Animals**

Quails (*C. coturnix* japonica), females, at 6 weeks old and average weight of 115 g, were allocated in specific cages for the species, separated and fed with ration diet (100 to 120 g/bird/day) and water *ad libitum* and went through a 15-day adaptation period. Afterward, they were infected experimentally by oral inoculation, with 4x10^4^ trophozoites. From the seventh day after infection, trophozoites were observed in the birds. These ones were examined daily, for 15 days, by wet mounting method and observed under optical microscope, to confirm the infection. The body weight of the quails was evaluated in the beginning and at the end of the experiment.

***In vivo*** **assay**

The birds were divided in seven groups (n=5): A (control–healthy), B (control infected), C (control TW 0.01%), D (NB 0,88mg/kg), E (MTZ 25 mg/kg), F (OE-CL at 0.55 mg/kg) and G (N-CL at 0.44 mg/kg). The treatments were managed orally (gavage) once a day, during 7 consecutive days (Youssefi et al., 2017).

**Committee of ethics**

The Committee of Ethics in the use of Animals of the Federal University of Pelotas, under the protocol 23110.005097/2017-51 and 23110.012860 / 2018-81 and SISBIO under number 61235-1 approved this research.

**Analyze statistical**

Statistical analysis was performed by univariate analysis of variance (ANOVA) using a probability value of p≤0.05, followed by the Tukey Test (GraphPad Prism 8.0 Software).

## Results

**Characterization** **OE-CL**

The chemical components of OE-CL was determined by gas chromatography. The major compounds were β-geranial (45.74%), Z-citral (34.42%) and geraniol (6.01%), respectively. 

**Characterization of** **N-CL**

All N-CL presented a polydispersity index lower than 0.3, indicating homogeneity of droplet size and characterizing a monodisperse system.espite the use of non-ionic surfactants in the formulation, the N-CLs had slightly negative zeta potential. 

N-CL is formed by mixing two immiscible liquids stabilized by surfactants, ranging in size from 20 μm to 500 nm. The N-CL developed in the present study had an average droplet size of less than 200 nm. The results of the characterization of N-CL with OE and white N-CL, according to mean size, polydisperasion index, zeta potential and pH, are shown in Table 1.

The under-refrigeration N-CLs were stable for 90 days as the parameters were maintained when compared to the parameters immediately after preparation. 

**Molecular results**

The *T. gallinae* isolate was confirmed by equivalence with the Genbank isolates. 

***In vitro*** **assay**

**Figure 1:**
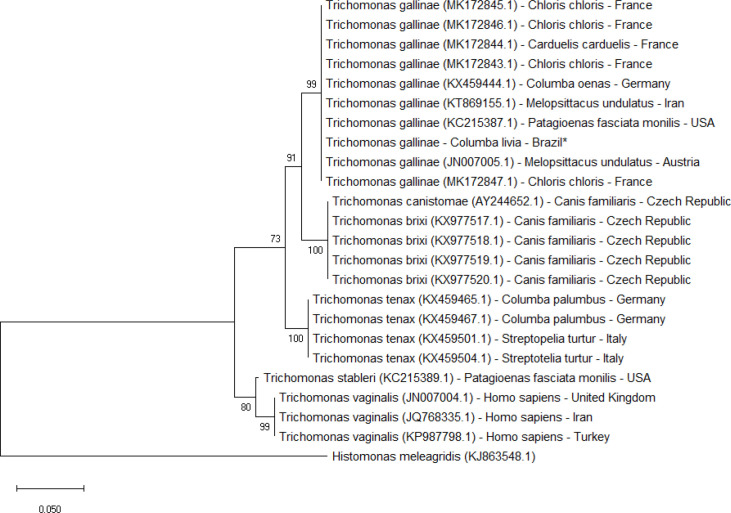
Phylogenetic analysis of the relationship based in ITS-1 rDNA sequences between *Trichomonas gallinae, T. brixi, T. canistomae, T. tenax, T. stableri* and *T. vaginalis*. Maximum likelihood tree (24 sequences and 336 sites). Bootstrap support (BS) was calculated for each branch using Mega X. *Histomonas meleagris* was the outgroup. Star indicates our isolate

The results of the *in vitro* study on the anti-trichomonal inhibitory effect of OE, N-CL and MTZ. The comparison of this activity with the CN (negative control) is presented in Figure 1. Neither OE-CL nor N-CL presented toxicity at the concentrations used in the study, being tested on other micro-organism.

For the N-CL after 24 hr incubation at 440 µg/ml concentration and 550 µg/ml for OE-CL, 100% unviable trophozoites in the culture medium was found (Figure 3). The MICs (minimum inhibitory concentration) of the free OE-CL and the N-CL were 528 and 418 µg/ml. IC_50_ values (minimum inhibitory concentration) were set at 110 µg/ml for N-CL and 220 µg/ml for free OE-CL. The growth of the trophozoites was completely inhibited by the free and nanostructured OE of lemon grass in 12 hr incubation (Figure 4). 

**Table 1:** Physical-chemical characterization of N-CL

	**Average droplet size (nm)**	**Polydispersity index**	**Zeta potential (mV)**	**pH**
N-CL	98±0.82	0.20±0.007	-7.48±1.02	4.00±0.07
Blank nanoemulsion	127±1.02	0.25±0.005	- 6.37±0.58	5.15±0.03

**Figure 2:**
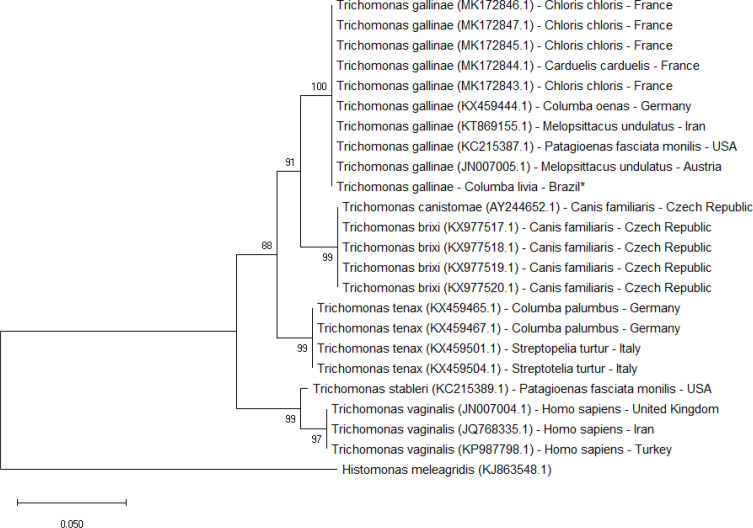
Phylogenetic analysis of the relationship based in SSU rDNA 18S sequences between *Trichomonas gallinae, T. brixi, T. canistomae, T. tenax, T. stableri and T. vaginalis*. Neighbour-Joining tree (24 sequences and 336 sites). Bootstrap support (BS) was calculated for each branch using Mega X. *Histomonas meleagris *was the outgroup. Star indicates our isolate

**Figure 3:**
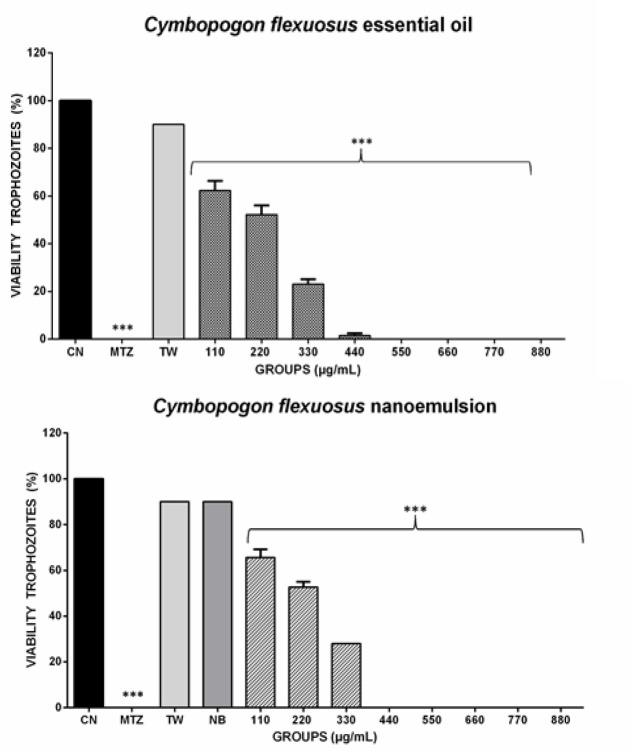
Anti-*trichomononas* activity in culture medium of the formulations: OE-CL and N-CL their respective at concentrations of 110 μg/mL to 880 μg/ml, as controls: CN (negative control), MTZ (metronidazole 880 μg/ml), (tween 0.01%) and NB (blank nanoemulsion 880 μg/ml). The analyses were done at 24 hr post-treatment. The columns indicate the groups and ***indicates the statistical difference when compared to the MTZ control by Tukey's test (p<0.05)

***In vivo*** **assay**

After the treatment with OE-CL trophozoites, the absence in 4-day post treatment birds (n=5) was observed, and until the 7th day, none of the birds of this group has presented viable forms of trophozoite in the swabs culture through 24 and 48 hr, comparing to all other group controls (p < 0.05). The treatment with N-CL presented efficiency on the 3^rd^ day of treatment with the trophozoites absence (n=5) after 4^th^ day of treatment, none of the birds of this group has presented viable trophozoites in the culture until the 7^th ^day. The MTZ presented trophozoites absence in the birds on the 7^th^ day of treatment. During the experiment deaths did not occur and there was no adverse effect observed in the treated animals. During the treatment (30 days), the body weight and laying of the treated quails and the groups controls have not shown changes (Figure 5).

**Figure 4:**
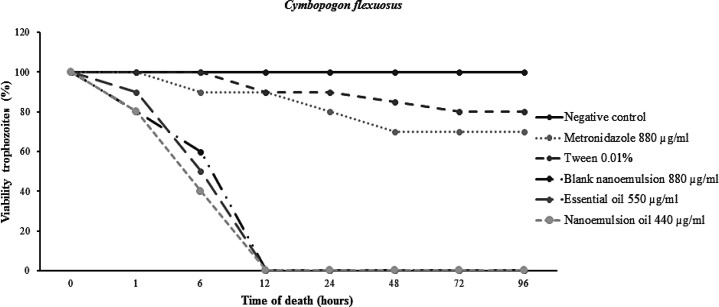
Death time of *T. gallinae* after treatment with negative control group, blank nanoemulsion control, OE-CL and N-CL, in the period of 1, 6, 12, 24, 48, 72 and 96 hr

## Discussion

Drugs like MTZ, dimetridazole, ronidazole and carnidazole, are considered efficient against trophozoites of *T. gallinae* (Franssen and Lumeij, 1992). The existence of resistant strains to the *in vivo* nitroimidazoles has already been demonstrated in several countries (Lumeij and Zwijnenberg (1990), Franssen and Lumeij (1992), Gerhold et al. (2008), Munoz et al. (1998), Rouffaer et al. (2014), Tabari et al. (2017), and threaten bird health, causing economic losses (Bondurant and Honigberg, 1994). This work is pioneer in studies related to OE-CL and N-CL on the *T. gallinae *flagellated protozoan in Brazil.

In our study, we used the lemon grass and in the gas chromatographic analysis, it was observed that β-geranial (45.74%), Z-citral (34.42%) and geraniol (6.01%) are the main constituents of OE. Adukwu et al. (2012) and Ahmad and Viljoen (2015) in their studies also identified Z-citral and β-geranial as main compounds of OE-CL, where they have found values of 33 to 32.9% of Z-citral and 47 and 46.1%, of β-Geranial, respectively, corroborating our results. Variations in the chemical constituents occur due to several factors: seasonality, temperature, water availability, among others (Xavier et al., 2016). The OE-CL trichomonicidal action in different concentrations could be related to the bioactive compounds of *C. flexuosus* that have antioxidant and fungicidal activities. One of the compounds is the citral (Silva et al. (2008; Shukla et al. 2009), that owns its ability to destroy the cell membrane integrity, releasing the cell compounds of *Geotrichum citri-aurantii* (Zhou et al., 2014) through a mechanism of cell membrane damage, compromising integrity and permeability (Tao et al., 2014).

Other natural compounds were efficient against Trichomonas spp. if compared to MTZ as presented in our study. Tabari et al. (2017) observed the efficiency of the OE *Pelargonium roseum* in pigeons, as on the 6^th^ day of treatment, trophozoites were not observed. In our study, the nanoemulsion presented similar results on the 3^rd^ day and the oil on the 4^th^ day of treatment. Youssefi et al. (2017) by analyzing the *in vitro* and *in vivo* effect of *Artemisia sieberi* , have found in the *in vivo* test that the treatment with OE at a dose of 50 mg/kg after 4 days led to the full recovery of the infected pigeons. The results obtained at the treatment with *C. flexuosus* free essential oil, corroborate with this study.

The N-CL used in this study has stability for a period of 90 days under refrigeration, when compared to the obtained parameters as soon as the preparation. Sonneville-Aubrun et al. (2004), have considered emulsion of an average droplet diameter (Z) less than 300 nm as nanoemulsion, corroborating our study, in which N-CL had a diameter less than 200 nm. Minor emulsions diameters are more stable (Mehlhorn et al., 2009).

**Figure 5:**
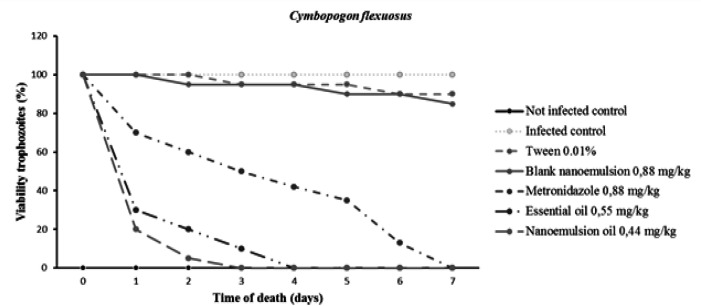
Death time of *T. gallinae* after treatment with the control groups and treatment groups with the standard drug, as well as, the OE-CL and N-CL in the period of 0.1. 2. 3. 4. 5. 6. 7 days

The stability is due to the use of surfactants in their formulation, increasing the availability and low interfacial tension penetration (Solans et al., 2005).

The greatest effectiveness (p<0.05) of nanoemulsion is compared to the essential oils in the *in vitro* and *in vivo* tests, could be attributed to the reduction of particle size, increase of bioavailability of chemical compounds and the surface charge of nanoemulsion droplets (potential zeta). However, in this study, there was not an electrostatic attraction, because N-CL has negative charges and the membrane of trophozoites is composed mainly of sialic acid, which also contributed to the protozoan negative charge (Costa e Souza, 1986). The relevance of researches with nanostructured systems with bioactives from plants is evident, as seen in works of Bahamond et al. (2015) and Wen-Chien et al. (2018). When the results of the standard drug used (MTZ) are compared, it has been different from the one in this study, which only presented the total elimination of the parasite from the 7th day of treatment. The OE-CL transport using N-CL is a new alternative to overcome limitations like hydrophobicity and volatility, reducing the drugs dose, and increasing the bioavailability and the potential effects of toxicity (Sun et al., 2012, Salem et al., 2015).

Nair et al. (2016) claimed that the therapeutic efficiency of many compounds, when associated to the nanotechnology, due to better solubilization, higher bioavailability and protection of the active principle against the enzymatic and hydrolytic degradation, occur. Our results showed a higher N-CL efficiency, which could be attributed to a greater penetration of nanoemulsion in the receptors present in the protozoan membrane, leading to a greater interaction between them. The higher the concentration of N-CL dose-dependent efficacy increases, occurring 100% of mortality in the greatest tested concentrations, demonstrating that the nanoparticles size (200 nm) increases the bioavailability, facilitating the EO activity (Trados et al. (2004).

In the *in vitro* test, the OE-CL and N-CL were able to reduce the viability of *T. gallinae. T. gallinae* trophozoites causing the cell death in different concentrations. The use of OE-CL was more effective against *T. gallinae* trophozoites with increasing concentrations of OE-CL and N-CL in the period of 12 hours, when compared to MTZ that took 24 hours. In the *in vivo* test, N-CL and OE-CL were more effective than the drug of choice, being able to eliminate the protozoan of the treated birds in 3 days for the N-CL use and 4 days for the OE-CL use, while MTZ only can eliminate the protozoan of all the treated birds after 7 days. The data obtained in this study introduced lemon grass like a natural anti-trichomonas agent, effective against trophozoites. The main chemical constituents of lemon grass can be considered like effective compounds for future researches and development of new anti-*T. gallinae* agents.

The isolated sample showed 99% similarity to the *T. gallinae* strains of *Acridotheres tristis*, Iran (KT869157.1), *Melopsittacus undulates*, Iran (KT869155.1), *Columbia livia*, China (MH733817.1), *Streptopelia decaocto*, Malta (KX844991.1), *Columba oenas*, Germany (KX459445.1), *Bubo bubo, *Spain (KX514378.1), *Serinus canaria* f.* domestica*, Slovenia (KX584000.1) and *Aquila pennata, *Spain* (*KP900042.1). On the other hand, the analysis among *Trichomonas* species, through ITS-1 rDNA (Figure 1) and SSU rDNA 18S (Figure 2), *Trichomonas gallinae* is in sister position of *T. canistomae* and *T. brixi*, both isolate of oral cavity from dogs and more distant of *T. vaginalis*. 
